# The Use of Bone Wax in Hemostatic Control for Total Knee and Hip Arthroplasties: A Systematic Review

**DOI:** 10.3390/jcm13102752

**Published:** 2024-05-07

**Authors:** Kenny Do, Benjamin Vachirakorntong, Eric Kawana, Jenifer Do, Thinh Dat Phan, Thinh Dai Phan

**Affiliations:** 1 Kirk Kerkorian School of Medicine, University of Nevada, Las Vegas, NV 89106, USA; kawana@unlv.nevada.edu; 2 College of Osteopathic Medicine, Touro University Nevada, Henderson, NV 89014, USA; bvachira2@student.touro.edu; 3 School of Life Sciences, University of Nevada, Las Vegas, NV 89154, USA; doj4@unlv.nevada.edu; 4 Pham Ngoc Thach University of Medicine, Ho Chi Minh City 700100, Vietnam; 1651010606@pnt.edu.vn (T.D.P.); 1651010607@pnt.edu.vn (T.D.P.); 5 Department of Internal Medicine, 115 People’s Hospital, Ho Chi Minh City Quận 10, Vietnam

**Keywords:** bone wax, hemostasis, knee arthroplasty, hip arthroplasty, orthopedic surgery

## Abstract

**Background/Objectives:** Blood loss can be a serious complication in patients undergoing total hip arthroplasty (THA) or total knee arthroplasty (TKA). Various methods are used by surgeons to achieve hemostatic control in these patients. Complications are associated with perioperative blood loss. In this systematic review, we examined the efficacy of using bone wax to control bleeding in patients undergoing THA and TKA. **Methods:** The PRISMA model was used to systematically identify and aggregate articles for this study. The PubMed and EMBASE databases were used to search individual studies that examined the use of bone wax in THA or TKA. After applying the search term “bone wax”, 2478 articles were initially identified. After inclusion and exclusion criteria were applied, three articles were aggregated for this systematic review. **Results:** The use of bone wax in THA and TKA decreased blood loss in patients undergoing these operations. Postoperative blood loss following surgery was lower in the bone wax groups compared to the control groups as well. Patients in the bone wax groups also required fewer blood transfusions than those who did not receive bone wax. **Conclusions:** Bone wax appears to be another modality that can be used by physicians to maintain hemostatic control in THA or TKA patients. Reduced blood loss and transfusion rates in surgery can increase patient outcomes. More studies are needed to examine the efficacy of bone wax in comparison with other hemostatic tools.

## 1. Introduction

Hemorrhagic complications during knee and hip surgery pose significant risks to patients because of the large amounts of bone and tissues involved during arthroplasty [1]. Bones contain a plethora of channels for vascularization, where bleeding may occur from the exposed cancellous bone after osteotomy [2]. By 2030, it is believed that 3.78 million total knee arthroplasties (TKA) and 572,000 total hip arthroplasties (THA) will be performed, which is a 673% and 174% increase from 2005 for the two procedures, respectively [3]. During these surgeries, patients may experience visible and hidden blood loss, where one study reported that the average total blood loss during total knee arthroplasty was 1474 mL, with half of the volume being hidden and lost into tissues [4]. Another study reported that the total blood loss for total hip arthroplasty was 1155 ± 377 mL, with the hidden blood loss being 35.4% ± 11.0% [5]. In rare and severe cases, significant blood loss during surgery may lead to hemorrhagic shock, hypovolemia, organ failure, thrombosis, and even death [6,7]. The current literature suggests that between 21% and 70% may require allogeneic blood transfusions when undergoing TKA and THA [8].

Because bone wax is hydrophobic and does not participate in biological reactions or shift the pH in the body, it acts as a great sealant to prevent further blood loss during knee or hip surgery [2]. The material is also easily applied to target the exposed trabecular bone, and its ability to stop bleeding is instantaneous [9]. This allows for reduced transfusion rates and possibly reduced intraoperative complications and postoperative hospital stays [10,11]. There are possible risks associated with perioperative blood transfusions, such as hemolytic reactions, transmission of infections, immune suppression, increased hospital stay, and even increased mortality [12,13]. Using bone wax not only reduces bleeding during surgery, but it may reduce perioperative blood transfusions as well, allowing for resources to not be wasted.

Various hemostatic techniques and agents can be used to reduce bleeding during surgery. These hemostatic agents can be categorized into four groups. There are biologically active agents (albumin, recombinant thrombin, fibrin sealants), synthetic sealants (polyethylene glycol hydrogel), hemostatic dressings (chitin, dry fibrin, kaolin), and physical/absorbable products (bone wax, porcine collagen, gelatin foams) [14]. One of the most effective and widely used hemostatic agents is tranexamic acid, an antifibrolytic agent that has been studied to reduce the need for perioperative blood transfusions [14]. Another hemostatic device that has been used to reduce bleeding during TKA and THA is bone wax, which is a topical product consisting of 70% beeswax and 30% Vaseline (soft paraffin) [15,16]. In this systematic review, we examined if the use of bone wax for patients undergoing TKA and THA reduces perioperative bleeding and the need for transfusions.

## 2. Methods

### 2.1. Study Design

This systematic review used the PRISMA model to identify and aggregate studies related to the use of bone wax to control hemostasis during hip and knee arthroplasties [17]. The PubMed (MED-LINE) and Excerpta Medica (Embase) databases were used to retrieve the articles. The broad search term “bone wax” was employed in these databases. Our search terms produced 416 articles on the PubMed database with years ranging from 1911 to 2023 and produced 2062 articles on the Embase database with years ranging from 1974 to 2023. This systematic review has not been registered in PROSPERO. The review protocol is available upon request. Furthermore, it is important to note that we did not perform statistical analysis for these results. We only aggregated, reported, and summarized the results found in each of the individual studies.

### 2.2. Study Selection

**Figure 1:**
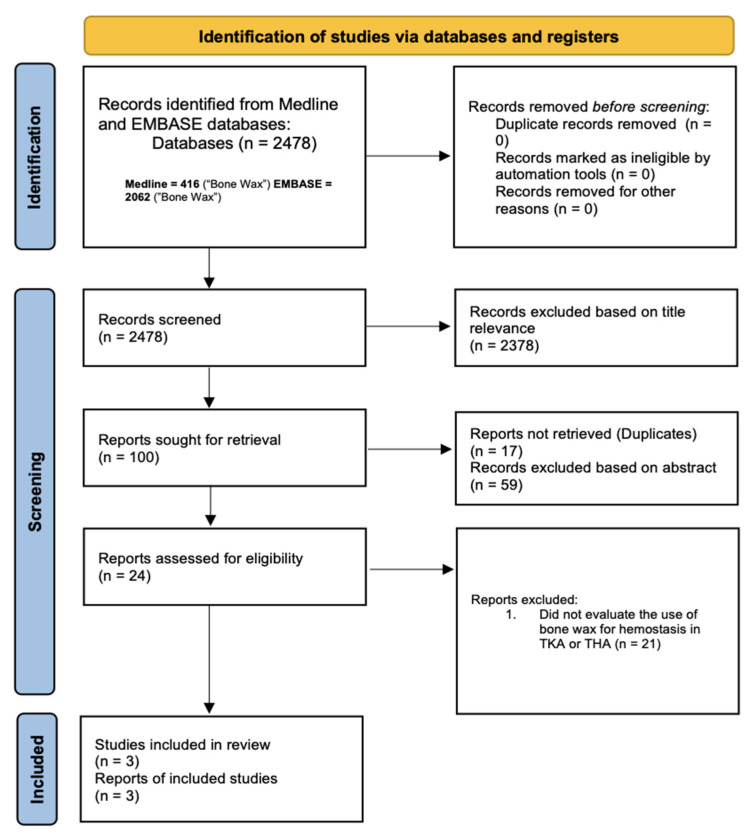
Study flow chart [17].

In accordance with the PRISMA review model, the titles of articles were examined for relevancy to this systematic review by three independent investigators [17]. After inputting the search term “bone wax” in the PubMed and Embase database, 2478 articles were identified. Out of these articles, only 100 studies had titles that were deemed relevant to the investigation. Articles were excluded during this stage if the titles did not mention orthopedic procedures, perioperative blood loss, hemostatic agents, or bone wax. Next, 17 duplicate papers were removed, and the abstracts of the remaining 83 articles were evaluated. After evaluation of the abstracts, 59 articles were removed. The investigators had more specific inclusion criteria for the abstracts, where articles were removed if their abstracts did not discuss hemostasis in TKA/THA, the use of bone wax in TKA/THA, or complications associated with the application of bone wax in orthopedic procedures. During the screening of titles and abstracts, articles were removed if they were not written in English or were not full-length article publications (i.e., abstract publications were excluded from this study). The last step in systematically screening the studies involved the evaluation of the remaining 24 article’s full-length papers. After evaluation, only 3 articles fit our inclusion and exclusion criteria. In this final stage, articles were only included if they specifically assessed the effectiveness of bone wax in controlling blood loss in TKA or THA. Articles were excluded if they focused on other hemostatic agents besides bone wax in controlling blood loss or did not focus on hip or knee arthroplasties. The steps of our PRISMA evaluation methods are displayed in Figure 1.

For this systematic review, the PICO inclusion criteria encompassed the following: 1. Population: adult or pediatric patients who underwent TKA or THA; 2. Intervention: applying bone wax to control blood loss in TKA or THA; 3. Control: patients who did not receive bone wax for hemostatic control; 4. Outcome: perioperative blood loss and blood transfusion rates; 5. Study design: observational studies or randomized control studies that assessed the use of bone wax in TKA or THA. Table 1 and Table 2 shows the demographics of the patients as well as their responses to the use of bone wax for blood loss.

### 2.3. Quality Assessment and Data Abstraction

The quality of each of the studies used for our systematic review was evaluated using a tool provided by the National Institute of Health [18]. The quality assessment tool was used for the randomized control studies, with a total of 14 questions, and another tool was used for the case–control studies, with a total of 12 questions. Moo et al. [9] fulfilled all 14 criteria, Mortazavi et al. [19] fulfilled 13 out of 14 criteria, and Shin et al. [10] fulfilled 11 out of 12 criteria. The questions used for evaluating the risk of bias are shown in Table 3 and Table 4 [18]. The risk of bias was determined based on the number of questions that received a “yes” response. If the majority of the responses were “yes”, then the study would be deemed to have a low risk of bias. All 3 articles had a low risk of bias based on the questionnaire and were therefore considered good studies.

## 3. Results

### 3.1. Study Characteristics

Our systematic search yielded a total of 3 articles that pertained to our investigation. A total of 456 patients were included in this systematic review, with 229 patients who were part of the control group and 227 patients who received bone wax during TKA or THA. The number of male patients in these studies was 120 and the number of female patients was 336. The average age of these patients who either received TKA or THA was approximately 65 years old. The demographics of the patients are demonstrated in Table 1.

**Table 1:** Demographics of bone wax and control groups [9,10,19].

Article	Study Type	Number of Patients Treated	Age	Gender
Mortazavi et al. [19]	Randomized control study	75 in bone wax 77 in control group	Mean age 47.7 ± 15.4 ranging from the ages 17–83	50.7% (77 patients) were Female 49.3% (75 patients) Male
Shin et al. [10]	Retrospective Case–Control Study	102 in bone wax group 102 in control group	72.13 ± 6.55 in bone wax group 72.78 ± 7.27 in control group	12 males and 90 females in bone wax group 9 males and 93 females in control group
Moo et al. [9]	Prospective randomized control study	50 patients in bone wax group 50 patients in control group	65.8 ± 8 in bone wax group 67 ± 7 in control group	14 males and 36 females in bone wax group 10 males and 40 females in control group

**Table 2:** Blood loss and transfusion rates in bone wax versus control groups [9,10,19].

Article	Study Type	Year		Number of Patients Control	Number of Patients Bone Wax	Blood Loss Wax (mL)	Blood Loss Control (mL)	Number of Transfusions Bone Wax	Number of Transfusions Control	Keen Findings	Risk of Bias Assessment	Risk of Bias Assessment Explanation
Moo et al. [9]	Prospective randomized control study	2016	TKA	50	50	508.2 ± 259.8	689.2 ± 282.7	0	3	Postoperative day 1 blood loss was lower in bone wax group than in the control group. Number of transfusions was lower in the bone wax group as well.	14/14	Good: Fulfilled all of the risk of bias criteria (Table 3)
Mortazavi et al. [19]	Randomized control study	2022	THA Direct Antioer Apporach	77	75	505.2 (409.2–637.6)	747.0 (494.6–955.4)	0 (0%)	2 (2.6%)	Blood loss and rate of transfusion were lower in the bone wax group than the control group. Postoperative blood loss on day 3 was lower in the bone wax group.	13/14	Good: Groups were not at the same baseline on characteristics that could affect outcome (Question 6 on Table 3)
Shin et al. [10]	Retrospective Case–Control Study	2020	TKA	102	102	458.29 ± 192.35	539.06 ± 310.51	2 (2.0)	9 (8.8)	Estimated blood loss was lower in the bone wax group than in the control group. Transfusion rates were lower in the bone wax group as well.	11/12	Good: Assessors of the exposure were not blinded to the group of the participant. (Question 11 on Table 4)

**Table 3:** Quality assessment of controlled intervention studies [18].

Criteria	Moo et al. [9]	Mortazavi et al. [19]
1. Was the study described as randomized, a randomized trial a randomized clinical trial, or an RCT?	Yes	Yes
2. Was the method of randomization adequate?	Yes	Yes
3. Was the treatment allocation concealed?	Yes	Yes
4. Were the study participants and providers blinded to treatment group assignment?	Yes	Yes
5. Were the people assessing the outcomes blinded to the participants’ group assignments?	Yes	Yes
6. Were the groups similar at baseline on important characteristics that could affect outcomes?	Yes	No
7. Was the overall drop-out rate from the study at endpoint 20% or lower of the number allocated to treatment?	Yes	Yes
8. Was the differential drop-out rate (between treatment groups) at endpoint 15 percentage points or lower?	Yes	Yes
9. Was there high adherence to the intervention protocols for each treatment group?	Yes	Yes
10. Were other interventions avoided or similar in the groups (e.g., similar background treatments)?	Yes	Yes
11. Were outcomes assessed using valid and reliable measures, implemented consistently across all study participants?	Yes	Yes
12. Did the authors report that the sample size was sufficiently large to be able to detect a difference in the main outcome between groups with at least 80% power?	Yes	Yes
13. Were outcomes reported or subgroups analyzed prespecified?	Yes	Yes
14. Were all randomized participants analyzed in the group to which they were originally assigned?	Yes	Yes

**Table 4:** Quality assessment of case–control Studies [18].

	Shin et al. [10]
1. Was the research question or objective in this paper clearly stated and appropriate?	Yes
2. Was the study population clearly specified and defined?	Yes
3. Did the authors include a sample size justification?	Yes
4. Were controls selected or recruited from the same or similar population that gave rise to the cases (including the same timeframe)?	Yes
5. Were the definitions, inclusion and exclusion criteria, algorithms or processes used to identify or select cases and controls valid, reliable, and implemented consistently across all study participants?	Yes
6. Were the cases clearly defined and differentiated from controls?	Yes
7. If less than 100 percent of eligible cases and/or controls were selected for the study, were the cases and/or controls randomly selected from those eligible?	Yes
8. Was there use of concurrent controls?	Yes
9. Were the investigators able to confirm that the exposure/risk occurred prior to the development of the condition or event that defined a participant as a case?	Yes
10. Were the measures of exposure/risk clearly defined, valid, reliable, and implemented consistently (including the same time period) across all study participants?	Yes
11. Were the assessors of exposure/risk blinded to the case or control status of participants?	No
12. Were key potential confounding variables measured and adjusted statistically in the analyses? If matching was used, did the investigators account for matching during study analysis?	Yes

### 3.2. Overview: Total Knee and Hip Arthroplasties

Out of the three articles included in our systematic review, two of them examined the use of bone wax for TKA and one examined the use of bone wax for THA. These articles included two randomized control trials and one retrospective case–control study. Moo et al. [9] and Shin et al. [10] studied the hemostatic use of bone wax in TKA and Mortazavi et al. [19] studied the use of bone wax in direct THA. Postoperative blood loss as well as transfusion rates were decreased across these three studies examining patients who underwent knee and hip surgery with the application of bone wax.

### 3.3. Total Knee Arthroplasty

Moo et al. conducted a prospective randomized controlled study that assessed the efficacy of applying bone wax for 100 patients who underwent unilateral TKA [9]. The arthroplasties were performed under the mini-midvastus approach with patella eversion method, where the ages of the patients ranged from 50 to 85. The bone wax and control groups were each randomly assigned 50 patients. Following their surgical procedures, all patients were provided with cemented prostheses. Subsequently, the treatment group underwent an additional procedure where 2.5 g of bone wax was firmly applied around the femoral and tibial prostheses, specifically targeting the exposed cancellous bone surface to effectively seal the nail holes. In this study, the researchers found that patients who received bone wax experienced a significant decrease in total blood loss and hemoglobin drop on the first postoperative day (POD). On the first POD, the bone wax group experienced a total blood loss of 508.2 ± 259.8 mL, which is significantly less than the 689.2 ± 282.7 mL experienced by the control group. Furthermore, the hemoglobin drop on the first POD was 1.6 ± 0.9 in the bone wax group as compared to 2.1 ± 1.1 in the control group. On the third POD, blood loss was 987.9 ± 341.7 mL for the bone wax group and 1183.5 ± 334.7 mL for the control group. It is important to note that tranexamic acid (TXA) was not used in any of the patients. None of the patients in the bone wax group needed blood transfusions, while three patients in the control group needed transfusions. No patients in either group experienced any serious complications [9].

The retrospective observational study by Shin et al. reviewed TKA operations using the cemented posterior cruciate-substituting design and resurfacing of the patella for osteoarthritis [10].

Bone wax was applied to the cancellous bone around the femoral and tibial prosthesis after implantation. The bone wax group (102 patients) had an average blood loss of 458.29 ± 192.35 mL and the control (102 patients) group’s average blood loss was 539.06 ± 310.51 mL, with each group having 102 patients. On the first POD, the peak reduction in hemoglobin level was 1.71 ± 0.75 mL in the bone wax group compared to 2.16 ± 0.98 mL in the control group. On the seventh POD, the peak reduction in hemoglobin levels was 2.90 ± 0.83 mL compared to 3.30 ± 1.13 mL in the control group. Blood loss and peak decline in hemoglobin levels were lower in the patients treated with bone wax compared to the control patients throughout the entire course of their postoperative recovery.

### 3.4. Total Hip Arthroplasty

Mortazavi et al. conducted a randomized controlled clinical trial consisting of 152 patients who underwent THA through the direct anterior approach [19]. The bone wax group consisted of 75 patients and the control group without bone wax consisted of 77 patients. The main objective of this triple-blinded study was to assess the efficacy of bone wax in reducing apparent and total perioperative blood loss (PBL) for patients undergoing THA. The bone wax was pasted onto the distal femoral neck after the operation for the targeted group. Similar to the TKA patients previously mentioned, the bone wax group had an apparent PBL of 200.0 (115.0–310.0) mL and the control group had an apparent PBL of 370.0 (195.0–513.7) mL. The total PBL on day 3 was 505.2 (409.2–637.6) mL for the bone wax patients and 747.0 (494.6–955.4) for the patients who did not receive bone wax. This trend was observed in the total PBL on day 5 as well. This study suggests that the blood loss in patients undergoing THA was statistically lower for those who received bone wax [19].

## 4. Transfusion Rates

In Moo et al., zero patients who underwent TKA in the bone wax group required transfusion, while three in the control group needed transfusions [9]. In Shin et al., two patients who underwent TKA in the bone wax group required transfusions, while nine in the control group required transfusion [10]. In Mortazavi et al., zero patients who underwent THA needed blood transfusions, while two in the control group required transfusion [19]. This demonstrates that not only does bone wax decrease perioperative bleeding, but it decreases the need for allogeneic blood transfusions in TKA and THA operations as well.

## 5. Discussion

Serious complications can arise from TKA and THA due to uncontrolled postoperative bleeding, which is often associated with prolonged rehabilitation and worsened mortality [19,20,21]. Blood loss is a serious complication experienced by patients undergoing these procedures, where one study reported that the overall transfusion rate was 11% in TKA patients and 18% in THA patients [8]. Uncontrolled blood loss during surgeries have been associated with longer hospital stays and costlier billing [22]. Complications of anemia following surgery may lead to infection, sepsis, slow healing of wounds, venous thromboembolism, stroke, and even cardiovascular problems [23]. Monsef et al. reported an increase in hospital length of stay in the number of allogeneic blood units that patients received post-surgery versus autologous blood [22].

Effective hematogenous tools have been demonstrated to decrease the need for transfusion rates. Tranexamic acid (TXA) is a common agent that inhibits plasminogen activity, thus preventing the elimination of fibrin clots [24]. Past studies have shown that patients receiving TXA are less likely to need transfusion, with one finding that only 7.1% of patients who received four doses of TXA needed blood transfusion, as compared to those who did not receive TXA [25]. An additional method for managing blood loss during arthroplasties involves employing bone wax, which operates through a different mechanism to halt bleeding. Depending on the individual surgical conditions and patient considerations, bone wax might be favored over TXA or utilized alongside it to attain the best possible hemostasis.

The use of bone wax is thought to have begun as early as the 1700s, but it was not until 1885 that Sir Victor Horsley popularized and concocted an ingredient that was widely used for blood loss during times of war [2,26]. This mixture consisted of a ratio of seven parts beeswax to one part almond oil, with one percent salicylic acid included [2,27]. However, it was not until 1892 that Rushton Parker applied bone wax for hemostatic control during surgery [2,28]. Today, the ingredient for bone wax is relatively the same, containing white beeswax and isopropyl palmitate [26].

Bone wax is most effective when used at temperatures between 21 and 23 °C [26]. Its malleable texture facilitates easy handling, enabling it to cover bone surfaces and halt active bleeding while promoting clot development. This property has made it an effective hemostatic tool in orthopedic surgeries, neurosurgery, craniofacial surgery, and more [26,29].

The individual articles aggregated in this systematic review report a decreased need for transfusions as well as a decrease in perioperative blood loss with the application of bone wax. Perioperative blood loss on days 1, 3, and 5 following the operations was lower in the bone wax group versus control. Out of the three studies analyzed in this systematic review, only one of them documented a single case of surgical site infection in a patient treated with bone wax. However, the hemostatic outcomes in these studies cannot be attributed to the use of bone wax alone. Various factors, including patient attributes, surgical complications, operative site, tourniquet use, cement application, TXA administration, and other variables, can influence hemostatic outcomes apart from the use of bone wax. Bone wax is an alternative tool that surgeons can consider for hemostatic control. Additional randomized controlled trials are required to enhance the medical community’s understanding of the effectiveness of bone wax when compared to alternative hemostatic methods, such as TXA.

## 6. Limitations

There are limitations to this study. First, adverse effects have been seen in patients who receive bone wax during surgery. Solomon et al. wrote a prospective case series that examined the side effects of bone wax when used intra-articularly to decrease bleeding during femoroacetabular impingement (FAI) surgery [30]. Fourteen patients who required revision surgery were included in the study, with eleven of them having received bone wax during their first operations. The researchers discovered that all eleven patients who had received bone wax revealed foreign body-type chronic synovial inflammation on histology, where five of them also had lymphoplasmacytic inflammatory reactions. Grossly, all of the 11 patients experienced swelling, engorgement, and inflammation of the synovial joints [30].

Another limitation of our study is the use of only two databases in searching for the articles for this systematic review. The PubMed and EMBASE databases were employed to retrieve these studies, and although the two databases are extensively comprehensive, there is a possibility that some were missed during our search. The systematic review’s aggregated articles may be susceptible to publication bias, as only studies demonstrating successful applications of bone wax in TKA or THA may have been published. Consequently, instances where bone wax was unsuccessful for hemostatic control could potentially be underreported. Future directions may include the use of more databases to potentially identify articles not indexed in PubMed or EMBASE.

## 7. Conclusions

Bone wax is another modality that can be used by orthopedic surgeons to control blood loss during hip or knee arthroplasties. This systematic review of the current literature demonstrates that bone wax can reduce blood loss as well as reduce transfusion rates during these operations. However, it is important to note that many factors should be considered when assessing the efficacy of bone wax in hemostatic control. For example, the use of TXA, tourniquet, patient medical history, and other variables in the individual studies may affect the true outcome of bone wax in controlling blood loss. More randomized control trials are needed to better understand the efficacy and safety of bone wax in comparison with other hemostatic methods. In the future, we may consider the addition of a meta-analysis to add statistical analysis to these findings. Furthermore, future studies may include the direct comparison of bone wax against other products, such as gelatin-thrombin matrix sealants, TXA, and more. We may consider examining the use of bone wax in other procedures as well, such as in lumbar laminotomy, orbital procedures, dermatologic surgeries, and more.

## Data Availability

Data are public information and are available upon request.

## References

[1] Prasad N., Padmanabhan V., Mullaji A. (2007). Blood loss in total knee arthroplasty: An analysis of risk factors. Int. Orthop..

[2] Zhou H., Ge J., Bai Y., Liang C., Yang L. (2019). Translation of bone wax and its substitutes: History, clinical status and future directions. J. Orthop. Transl..

[3] Kurtz S., Ong K., Lau E., Mowat F., Halpern M. (2007). Projections of primary and revision hip and knee arthroplasty in the United States from 2005 to 2030. J. Bone Jt. Surg. Am..

[4] Sehat K.R., Evans R., Newman J.H. (2000). How much blood is really lost in total knee arthroplasty? Correct blood loss management should take hidden loss into account. Knee.

[5] Miao K., Ni S., Zhou X., Xu N., Sun R., Zhuang C., Wang Y. (2015). Hidden blood loss and its influential factors after total hip arthroplasty. J. Orthop. Surg. Res..

[6] Rossi G., Mavrogenis A., Angelini A., Rimondi E., Battaglia M., Ruggieri P. (2011). Vascular complications in orthopaedic surgery. J. Long-Term Eff. Med. Implant..

[7] Kwon Y.S., Kim H., Lee H., Kim J.H., Jang J.S., Hwang S.M., Hong J.Y., Yang G.E., Kim Y., Lee J.J. (2021). Effect of Intra- and Post-Operative Fluid and Blood Volume on Postoperative Pulmonary Edema in Patients with Intraoperative Massive Bleeding. J. Clin. Med..

[8] Carling M.S., Jeppsson A., Eriksson B.I., Brisby H. (2015). Transfusions and blood loss in total hip and knee arthroplasty: A prospective observational study. J. Orthop. Surg. Res..

[9] Moo I.H., Chen J.Y.Q., Pagkaliwaga E.H., Tan S.W., Poon K.B. (2017). Bone Wax Is Effective in Reducing Blood Loss After Total Knee Arthroplasty. J. Arthroplast..

[10] Shin K.H., Choe J.H., Jang K.M., Han S.B. (2020). Use of bone wax reduces blood loss and transfusion rates after total knee arthroplasty. Knee.

[11] Le Huec J.C., AlEissa S., Bowey A.J., Debono B., El-Shawarbi A., Fernández-Baillo N., Han K.S., Martin-Benlloch A., Pflugmacher R., Sabatier P. (2022). Hemostats in Spine Surgery: Literature Review and Expert Panel Recommendations. Neurospine.

[12] Crosby E.T. (1992). Perioperative haemotherapy: II. Risks and complications of blood transfusion. Can. J. Anaesth..

[13] Elmi M., Mahar A., Kagedan D., Law C.H., Karanicolas P.J., Lin Y., Callum J., Coburn N.G., Hallet J. (2016). The impact of blood transfusion on perioperative outcomes following gastric cancer resection: An analysis of the American College of Surgeons National Surgical Quality Improvement Program database. Can. J. Surg..

[14] Behrens A.M., Sikorski M.J., Kofinas P. (2014). Hemostatic strategies for traumatic and surgical bleeding. J. Biomed. Mater. Res. A.

[15] Schonauer C., Tessitore E., Barbagallo G., Albanese V., Moraci A. (2004). The use of local agents: Bone wax, gelatin, collagen, oxidized cellulose. Eur. Spine J..

[16] Tham T., Roberts K., Shanahan J., Burban J., Costantino P. (2018). Analysis of bone healing with a novel bone wax substitute compared with bone wax in a porcine bone defect model. Future Sci. OA.

[17] Page M.J., McKenzie J.E., Bossuyt P.M., Boutron I., Hoffmann T.C., Mulrow C.D., Shamseer L., Tetzlaff J.M., Akl E.A., Brennan S.E. (2021). The PRISMA 2020 statement: An updated guideline for reporting systematic reviews. BMJ.

[18] U.S. Department of Health and Human Services (2021). Study Quality Assessment Tools.

[19] Mortazavi S.M.J., Razzaghof M., Ghadimi E., Seyedtabaei S.M.M., Vahedian Ardakani M., Moharrami A. (2022). The Efficacy of Bone Wax in Reduction of Perioperative Blood Loss in Total Hip Arthroplasty via Direct Anterior Approach: A Prospective Randomized Clinical Trial. JBJS.

[20] Goker B., Caglar O., Kinikli G.I., Aksu S., Tokgozoglu A.M., Atilla B. (2022). Postoperative bleeding adversely affects total knee arthroplasty outcomes in hemophilia. Knee.

[21] Di Francesco A., Flamini S., Fiori F., Mastri F. (2013). Hemostatic matrix effects on blood loss after total knee arthroplasty: A randomized controlled trial. Indian J. Orthop..

[22] Monsef J.B., Della Valle A.G., Mayman D.J., Marx R.G., Ranawat A.S., Boettner F. (2014). The Impact of Blood Management on Length of Stay After Primary Total Knee Arthroplasty. Open Orthop. J..

[23] Kunz J.V., Spies C.D., Bichmann A., Sieg M., Mueller A. (2020). Postoperative anaemia might be a risk factor for postoperative delirium and prolonged hospital stay: A secondary analysis of a prospective cohort study. PLoS ONE.

[24] Haratian A., Shelby T., Hasan L.K., Bolia I.K., Weber A.E., Petrigliano F.A. (2021). Utilization of Tranexamic Acid in Surgical Orthopaedic Practice: Indications and Current Considerations. Orthop. Res. Rev..

[25] Kahan J.B., Morris J., Li D., Moran J., O’Connor M.I. (2021). Expanded use of tranexamic acid is safe and decreases transfusion rates in patients with geriatric hip fractures. OTA Int..

[26] Das J.M. (2018). Bone Wax in Neurosurgery: A Review. World Neurosurg..

[27] Ellis H. (2007). Horsley’s wax. J. Perioper. Pract..

[28] Gupta G., Prestigiacomo C.J. (2007). From sealing wax to bone wax: Predecessors to Horsley’s development. Neurosurg. Focus.

[29] Nooh N., Abdullah W.A., Grawish M.E.A., Ramalingam S., Javed F., Al-Hezaimi K. (2014). The effects of surgicel and bone wax hemostatic agents on bone healing: An experimental study. Indian J. Orthop..

[30] Does Bone Wax Induce a Chronic Inflammatory Articular Reaction?. https://www.ncbi.nlm.nih.gov/pmc/articles/PMC3462874/.

